# Coupling Signals between the Osteoclast and Osteoblast: How are Messages Transmitted between These Temporary Visitors to the Bone Surface?

**DOI:** 10.3389/fendo.2015.00041

**Published:** 2015-03-24

**Authors:** Natalie A. Sims, T. John Martin

**Affiliations:** ^1^Department of Medicine, St. Vincent’s Institute of Medical Research, St. Vincent’s Hospital, The University of Melbourne, Fitzroy, VIC, Australia

**Keywords:** osteoblast, osteoclast, coupling, osteocyte

Skeletal mass is regulated by two key activities: bone removal (resorption) by hematopoietic lineage osteoclasts and bone matrix formation by mesenchymal lineage osteoblasts. During adult life, these activities occur sequentially on the same surface: a process termed as remodeling. Tiny packets of bone are removed by osteoclasts and replaced by new bone matrix produced by osteoblasts. This continual renewal process allows repair of mechanical imperfections and calcium homeostasis.

The group of cells responsible for remodeling is termed as the basic multicellular unit (BMU) (1). To maintain bone mass at the same level during adulthood, the bone formed in each BMU must replace precisely the amount removed by resorption within that BMU. This stimulation of osteoblast activity in response to resorption is termed “coupling” (2), and it has long been of interest to understand how these two distinct cell types, on the same bone surface but at different times, could be linked so their activities are equal. The BMU and coupling concepts originally included only osteoclasts and osteoblasts, but over recent years, as more cellular contributors to remodeling have been identified (such as T-cells, macrophages, osteocytes, and precursor populations of osteoblasts and osteoclasts), the number of cells in the BMU has expanded (3–5). So too, more signaling pathways within the BMU have been identified (6, 7). All these signals converge on two cell types: the osteoclast and osteoblast, for only those cells are able of bone resorption and bone formation, respectively.

Osteoclasts and osteoblasts are not present on the bone surface simultaneously; the BMU exists, in different forms, at the same location over approximately 6 months in human bone. Early studies using undecalcified bone histology and timed fluorochrome labeling identified that bone resorption in iliac crest trabecular BMUs of adult human bone takes approximately 3 weeks (8), the formation response 3–4 months (9), and between the two activities there is a poorly understood “reversal phase” (10) of approximately 5 weeks (8). In rodents, the duration of this sequence is compressed, but a time delay between resorption and formation still exists: in rat alveolar bone, the reversal phase lasts for approximately 3.5 days (11). These numbers vary also with site, skeletal health, and treatment (12) and in some conditions, including osteoporosis there is an increased duration, or even arrest, of the reversal phase (13, 14). This review will explore mechanisms by which coupling signals may overcome the time delay between bone resorption and bone formation.

## The Main Classes of Coupling Factors

There are four main classes of osteoclast-derived signals that may promote bone formation in the BMU: (1) matrix-derived signals released during bone resorption, (2) factors synthesized and secreted by the mature osteoclast, (3) factors expressed on the osteoclast cell membrane, and (4) topographical changes effected by the osteoclast on the bone surface. We have reviewed these different proposed signals extensively elsewhere (6, 7), but will here focus on how each type of signal might influence bone formation after the reversal phase. As it has been noted in economics, there tends to be a proliferation of putative factors that determine process outcomes until they resemble a “zoo of factors,” with only a proportion of them being reliably reproduced in later research (15, 16). Since many coupling factors have only been identified in the past 10 years, most require validation by independent groups of researchers, working in multiple systems. This is particularly true of those factors identified in co-culture studies that disregard the time delay between bone resorption and formation.

### Matrix-derived factors

The bone matrix contains a store of latent growth factors, including transforming growth factor β (TGF-β), bone morphogenetic protein 2 (BMP-2), platelet-derived growth factor (PDGF), and the insulin-like growth factors (IGFs) (17–21). All are deposited by osteoblasts during matrix production then released by osteoclastic activity on the bone surface (20), as well as via plasminogen activators (22, 23) and matrix-metalloproteinases (24). These factors, once released from the matrix, are unlikely to remain within the bone microenvironment for some 5–8 weeks during the reversal phase until they can influence mature osteoblasts upon their arrival at the bone surface. Rather, their main influences would be to stimulate osteoblast progenitors, including their recruitment (25–27), migration (26, 28–30), and differentiation (29, 31, 32).

### Osteoclast-secreted factors

Osteoclasts also secrete products to promote osteoblast precursor recruitment and differentiation and thereby promote bone formation in the BMU (3). Many such “osteoclast-derived coupling factors” have been identified, and some were validated by studies of genetically altered mice: cardiotrophin-1 (33), sphingosine-1-phosphate, Wnt 10b, BMP-6 (34), CTHRC1 (35), and complement factor 3a (C3a) (36). We have described each of these in detail elsewhere (7); importantly, none are produced exclusively by osteoclasts, and many are also produced by other cell types in the vicinity of the BMU. Both active and inactive osteoclasts produce osteoclast-derived coupling factors (37). This concept is clinically important, since anti-resorptive inhibitors of osteoclast activity rather than osteoclast generation, such as cathepsin K inhibitors, may reduce bone resorption without blocking bone formation, leading potentially to an anabolic effect (38). Considering the time delay between bone resorption and formation, these factors cannot be expected to exist in a stable form at the BMU throughout the reversal phase. These factors stimulate not only the differentiated osteoblast but also, in the case of sphingosine-1-phosphate and cardiotrophin-1, stimulate precursor commitment to the osteoblast lineage (33, 34).

### Osteoclast membrane-bound factors

It has also been suggested that osteoclasts interact directly via cell surface regulatory proteins with mature osteoblasts to promote their activity. Factors proposed to act in this cell contact-dependent manner include EphrinB2 (39) and Semaphorin D (40). While plausible *in vitro* such cell contact-dependent mechanisms are problematic at the BMU because osteoclasts and osteoblasts are rarely in direct contact during remodeling. If such mechanisms occur, they may exist between osteoclasts and osteoblast precursors in the bone marrow space, or between osteoclasts and osteoblast-lineage cells in the remodeling canopy (41, 42), an anatomical structure observed above the BMU in human samples (43).

## Which Cell Type Responds to These Osteoclast-Derived Messages?

### Osteoblast precursors

Whether matrix-released, osteoclast-secreted, or membrane-bound, the osteoblast precursor seems a more likely target for coupling factors than the mature osteoblast. Indeed, mouse genetic experiments indicate that active TGF-β release during bone resorption induces osteoblast precursor migration to prior resorptive sites (29), thus making them available within the BMU for additional differentiation-promoting signals, such as matrix-derived IGF-1 (27). Osteoblast differentiation would be determined by these subsequent events, which are not well understood and may originate from a wide range of cells near the remodeling surface, including macrophages, T-cells, or the vasculature [reviewed in Ref. (7)]. Indeed, osteoclast-derived coupling factors are released by these other cell populations. For example, TGF-β, Semaphorin 4D, Wnt10b, and BMP-2 are released by T-cells (44–46) and macrophages (47, 48), while sphingosine-1-phosphate and PDGF are released by endothelial cells (49, 50). Nevertheless, the delay between osteoclast- or matrix-derived coupling factor release and the arrival of osteoblasts on the bone surface (5–8 weeks) is much longer than the time required for osteoblasts to differentiate *in vitro*. Murine stromal cell lines *in vitro* require only 7 days before alkaline phosphatase production commences (51) and <21 days until they express the genes associated with a matrix-embedded osteocyte (52, 53). Recent lineage tracing experiments suggest the process is even faster *in vivo*, with only 6 days passing before α-SMA precursors are incorporate into the bone matrix as osteocytes (54).

Osteoblast precursors, without the aid of any accessory cell, also sense the size and shape of pits formed by osteoclasts and preferentially form bone matrix in those locations (55). Osteoblast precursors respond to changes in surface topography, whether the change is much larger than the cell itself, as occurs with osteoclastic activity, or very much smaller than the cell (56). Altered nanotopography induces osteoblast filopodia formation followed by cytoskeletal changes involving cell adhesion and differentiation. However, whether osteoblasts preferentially form matrix on disrupted bone because they truly “sense” a change in physical dimensions of the bone or because they detect a change in surface composition is unclear; both scenarios are plausible. However, it is unlikely that the existence of a resorbed space provides all the information required for osteoblasts to initiate and complete refilling of the space with no input from other cell types. If that were true, remodeling would always be balanced and bone mass constant even in the presence of alterations in intercellular signals.

### Reversal cells

Another cell type that may respond to coupling factors are the reversal cells residing on the bone surface between the bone formation and resorption phases (10). These may provide both a physical and a temporal connection between the osteoclast and the osteoblast. They may respond to osteoclast-derived coupling factors (57), and pass the necessary signals to the osteoblast precursors as they move onto the bone surface. Interrogating this possibility remains a challenge, since the reversal cells lack specific identifying features that can be targeted by genetic or pharmacological means.

### Remodeling canopy

The remodeling canopy is another possible target for coupling factors (57). This anatomical structure consists of osteoblast-lineage cells that lift from the bone surface when osteoclastic resorption initiates the remodeling cycle (43). It has been suggested that the canopy is required for completion of the reversal phase, since biopsies from postmenopausal and glucocorticoid-induced osteoporotic patients exhibit incomplete canopies at sites of reversal phase arrest (58, 59). The canopy could also provide a controlled locale in which osteoblast-lineage cells, osteoclasts, and other contributing marrow cells, may exchange factors and influence precursors provided by the associated vasculature (41, 42). The bone remodeling canopy might serve as a way to keep local coupling factor concentrations sufficiently high to allow mesenchymal stem cell recruitment and to stimulate bone formation, or may limit the cellular contributors to the coupling processes. Defining the canopy’s contribution to the coupling process using genetically altered mouse models is limited because this anatomical structure has not been observed in the mouse, the model used most extensively for defining intercellular signaling pathways involved in bone remodeling.

### Osteocytes

Is there a role for the osteocyte in transmitting the messages from osteoclast to osteoblast in the BMU? Osteocytes are osteoblast-lineage cells trapped in the bone matrix during bone formation. They form an extensive interconnected network within a fluid-filled canalicular system (60), which may sense and respond to mechanical strain, as well as paracrine and endocrine signals (61). Osteocytes are present within the BMU throughout remodeling, and may therefore provide a system to transfer information from the resorbing osteoclast to bone surface osteoblasts. While few osteoclast-derived “coupling factors” have been shown to directly influence osteocytes, CT-1 reduces expression of sclerostin (52). CT-1 released by osteoclasts might therefore enter the lacunar–canalicular network, and act on those osteocytes closest to the resorptive site. When mature osteoblasts arrive on the bone surface, if sclerostin expression in the local area is still suppressed, this would allow bone formation to occur in this area. The factors that stimulate osteocytes to promote bone formation at resorbed sites may not be limited to CT-1. The mechanical disturbance caused by resorption itself may also “activate” osteocytes, and stimulate them to provide messages that promote bone formation on the same surface. One key question that remains is whether such a paracrine signaling mechanism could overcome the time delay between resorption and formation. For how long can osteocytes “retain” such information?

A mechanism that would not require a delayed communication system involving osteocytes is their detection and response to changes in microstrain that would occur during the remodeling process. Osteocytes would sense not only the increased strain resulting from weakening of the bone as resorption progresses (62) but would also detect when the strain is relieved as the bone is rebuilt by osteoblasts. Such a strain-based model for coupling was proposed some years ago (63) and now that our understanding of osteocyte signaling has improved, possible mediators are now coming to light. For example, sclerostin may mediate this process: sclerostin is significantly reduced by mechanical loading, and increased during unloading (64). Osteocytes may provide the final refining control to ensure that sufficient bone is formed by osteoblasts, generated in response to messages from osteoclasts either directly or via other cells within the BMU.

## Concluding Comments

**Figure 1:**
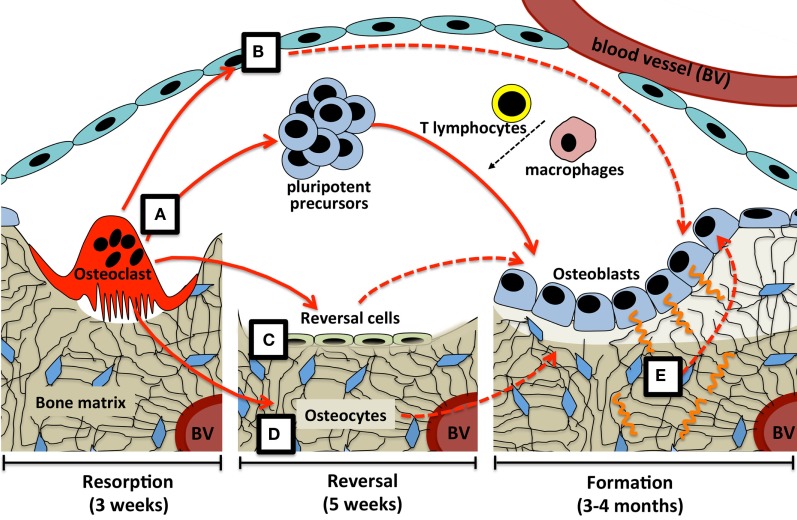
**Possible coupling mechanisms that overcome the time delay between bone resorption and formation**. **(A)** Osteoclast-derived factors (either released from the matrix, secreted from the osteoclast, or expressed on the osteoclast cell membrane) initiate differentiation of very early osteoblast progenitors, with the level of osteoblast activity and numbers of differentiated cells being refined by other factors released by a range of cell types within the BMU. Osteoclast-derived factors may act directly on cells that would transmit further signals (dashed lines) to both osteoblast precursors and mature osteoblasts; these transmitting cells could include **(B)** osteoblast-lineage cells in the remodeling canopy, **(C)** reversal cells on the bone surface, and **(D)** osteocytes. Finally, **(E)** physical changes brought about by the osteoclast, including the resorptive pit itself, and mechanical strain detected by the osteocyte network, would provide signals required for initiation and completion of the correct level of matrix production by mature osteoblasts on the bone surface. Periods of time required for each step are based on adult human iliac crest biopsies.

In conclusion, this suggests three mechanisms by which osteoclast-derived coupling signals may overcome the time delay between bone resorption and formation at the BMU (Figure 1). Osteoclast-derived factors (either released from the matrix, secreted from the osteoclast, or expressed on the cell membrane) initiate differentiation of very early osteoblast progenitors, with the level of osteoblast activity and numbers of differentiated cells being refined by other factors released by a range of cell types within the BMU. Second, osteoclast-derived factors may act directly on cells that would transmit further signals to both osteoblast precursors and mature osteoblasts; these transmitting cells could include reversal cells, osteoblast-lineage cells in the remodeling canopy, and osteocytes. Finally, physical changes brought about by the osteoclast would provide osteocytic signals required for initiation and completion of the correct level of matrix production by mature osteoblasts on the bone surface.

## Conflict of Interest Statement

The authors declare that the research was conducted in the absence of any commercial or financial relationships that could be construed as a potential conflict of interest.
